# Targeting PAR1 activation in JAK2V617F-driven philadelphia-negative myeloproliferative neoplasms: Unraveling its role in thrombosis and disease progression

**DOI:** 10.1016/j.neo.2025.101153

**Published:** 2025-03-14

**Authors:** İldeniz USLU-BIÇAK, Meliha NALÇACI, Selçuk SÖZER

**Affiliations:** a Department of Genetics, Aziz Sancar Institute of Experimental Medicine, Istanbul University, Istanbul, Türkiye; b Institute of Health Sciences, Istanbul University, Istanbul, Türkiye; c Department of Internal Medicine, Division of Hematology, Faculty of Medicine, Istanbul University, Istanbul, Türkiye

**Keywords:** Myeloproliferative Neoplasms, *JAK2*V617F, PAR1, PAR1 antagonist

## Abstract

Philadelphia chromosome-negative myeloproliferative neoplasms (Ph^-^MPNs) are clonal disorders marked by high morbidity and mortality, driven by uncontrolled myeloid proliferation from hematopoietic stem/progenitor cells (HSCs) and associated with a significant risk of thrombosis. This study explored the relationship between *JAK2*V617F and protease-activated receptor 1 (PAR1) by examining *PAR1* expression and activation across various hematopoietic stem/progenitor cell (HSPC) subgroups, assessing their contribution to the hypercoagulable state in Ph^-^MPNs.

We investigated the effects of thrombin, a PAR1 antagonist (vorapaxar), and a JAK2 inhibitor (ruxolitinib) on Ph^-^MPN cells. Mononuclear cells (MNCs) were isolated from Ph-MPN patients (*n* = 18), cord blood (CB) samples (*n* = 5) and healthy volunteers (*n* = 11). Specific subpopulations were sorted and analyzed for PAR1 expression and *JAK2*V617F status using qRT-PCR. *PAR1* expression changes, along with other PAR pathway-related genes, were assessed post-treatment.

Our results revealed that most PAR1^+^ cells (∼95 %) co-expressed CD34^+^, with a smaller JAK2V617F^+^ PAR1^+^ population lacking CD34. PAR1 expression was significantly higher in Ph-MPN MNCs compared to CB (*p* = 0.0005), particularly in EMP, HSC/EPC, and EPC subsets. Thrombin treatment reduced surface PAR1 expression, while PAR1 antagonist treatment further decrease the expression level. Combined PAR1 antagonist and ruxolitinib treatment significantly downregulated *PAR1* expression (*p* < 0.0001), and several PAR-pathway-related genes were notably downregulated after treatment.

This study highlights that elevated PAR1 expression in primitive hematopoietic subpopulations is linked to disease progression and thrombosis in Ph^-^MPNs, suggesting PAR1 as a potential therapeutic target. Combining PAR1 antagonists with JAK2 inhibitors shows promise in reducing PAR1 expression and mitigating thrombotic events in Ph^-^MPN patients.

## Graphical abstract

**Key findings of our study include:**

● **Elevated PAR1 Expression:** We identified significantly increased PAR1 expression in primitive hematopoietic subpopulations of Ph-MPN patients compared to controls.

● **PAR1 Activation and Thrombosis:** Our results suggest that PAR1 activation contributes to the hypercoagulable state observed in Ph-MPNs, offering a new avenue for therapeutic intervention.

● **Therapeutic Implications:** We demonstrated that the combination of PAR1 antagonists and JAK2 inhibitors effectively reduces PAR1 expression and downregulates thrombo-inflammatory pathways, indicating potential for improved clinical outcomes.

**Figure:**
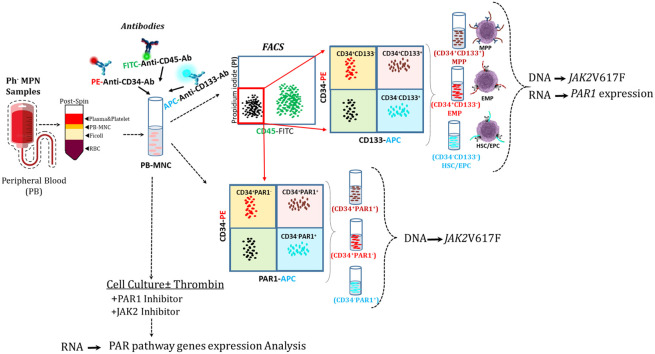

## Introduction

Philadelphia–negative myeloproliferative neoplasms (Ph^-^ MPNs) are a heterogeneous group of hematopoietic stem cells (HSC) originated disorders characterized by a range of clonal hematological diseases with overlapping clinic-pathological features including overproduction of blood cells in the bone marrow [1]. The main entities of MPNs, Polycythemia Vera (PV), Essential Thrombocythemia (ET), and Primary Myelofibrosis (PMF) are frequently reveal a genetic mutation, *JAK2*V617F which involves a change in the Janus kinase 2 (*JAK2*) gene, c.1849G>*T*, leading to the constitutive activation of the Janus kinase-signal transducer and activator of transcription (JAK/STAT) signaling pathway. The persistent activation of the pathway has been linked to enhanced cell survival, proliferation, and cytokine production, contributing to the pathogenesis of Ph^-^MPNs [2]. Ph^-^ MPNs has been described as a "human model of inflammation" which leads to atherosclerosis, other inflammatory diseases, secondary cancers, and a propensity for thrombotic events [[3], [4], [5], [6], [7], [8], [9]]. Given this, it is not unexpected that thrombosis is a common feature of MPN and significantly contribute to morbidity and mortality in affected individuals [[10], [11], [12], [13], [14]].

Protease Activated Receptors (PAR), are a family of G protein-coupled receptors including PAR1, PAR2, PAR3 and PAR4 [15] that play a role in the regulation of various physiological processes, including blood clotting and inflammation [16,17]. The ligand-receptor complex formed by proteolysis can activate different PAR molecules and induce different intracellular signaling. The three of PARs (PAR1, PAR3, and PAR4) are substrates for thrombin, a major serine protease involved in the coagulation cascade. Activation of PAR1 by thrombin triggers cleavage of the N-terminal extracellular domain of PAR1, and the new N-terminus acts as a tether ligand by binding intra-molecularly to the receptor itself, interacting with a specific region on the receptor surface [18] and initiating signaling [19,20] for its agonist activity [21].

Thrombin is the most potent known procoagulant [22] and studies have shown that thrombin-mediated PAR1 activation promotes the growth and invasion of cancer cells as well as hematopoiesis and HSC function [23,24] and in hematopoiesis and regulating HSC function [16,17]. Excessive thrombin infusions have been shown to induce HSC exit from the bone marrow (BM) via PAR1 activation on stromal cells and/or HSCs [25]. Interestingly, studies have shown that the endothelial protein C receptor (EPCR)/PAR1 signaling axis impacts HSC function [26], with differential regulation of HSC quiescence and BM retention [27].

In MPNs, the relationship between *JAK2*V617F allele burden and thrombin generation is well documented [[28], [29], [30]]. Thrombin levels are found to be higher among patients with PV and ET compared with individuals without MPN [31] and the presence of *JAK2*V617F in ET patients leads to increased platelet-derived thrombin production providing downstream regulation of several genes involved in thrombin generation [[28], [29], [30]]. Thrombin production has been observed as a biomarker of thrombotic risk in MPNs [32].

In this context, PAR1, emerges as a potential mediator in the complex network of signaling events associated with MPNs. However, the precise role of thrombin induced PAR1 activation in the context of *JAK2*V617F-driven MPN pathology remains largely unexplored. Given that the pathogenesis of MPN is known to originate from HSCs, the possibility that the manifestations of pro-inflammation may also have originated from HSCs. Therefore, in this study, the activation dynamics of the *PAR1* gene in HSCs harboring the *JAK2*V617F was studied. Initially, the study explored PAR1 activation in the peripheral blood hematopoietic progenitor stem cells (HPSC) subgroups of MPN patients and its relationship to *JAK2*V617F. Subsequently, the molecular profile of MPN cells was examined after specifically targeting thrombin induced PAR1 activation with PAR1 antagonist (vorapaxar), a synthetic tricyclic 3-phenylpyridine analog derived from natural product himbacine [33]. Vorapaxar selectively disrupts thrombotic pathways without impacting fibrinogen cleavage or fibrin formation, which are crucial for normal coagulation processes. Thus, it preserves the overall coagulation cascade and maintains normal bleeding times [34]. This targeted inhibition is especially beneficial in managing thrombotic complications in MPNs, where it is imperative to reduce thrombosis without elevating the risk of bleeding. Further studies were conducted using the JAK2 inhibitor, ruxolutinib, to enhance our understanding of PAR1 inhibition in MPN cellular processes.

Understanding the interplay between *JAK2*V617F-driven signaling, PAR1 activation, and the subsequent effects of PAR1 inhibition on HPSCs could offer valuable insights into the development of novel therapeutic strategies for MPNs. We demonstrated a significant increase in *PAR1* expression in HPSC subgroups, particularly in association with PAR1 inhibition, which altered the inflammatory state of MPN cells and mitigated the aberrant activation associated with MPN pathogenesis. By investigating the molecular underpinnings of PAR1 in this context, this study contributes to advancing precision medicine approaches, particularly through the application of target-specific PAR1 inhibitors that address the root causes of inflammation in MPNs, especially within the HPSCs.

## Materials and methods

### Patients and Samples

Eighteen patients who met the revised diagnostic criteria of the WHO 2008 for MPN [35], eleven healthy volunteers and five cord blood (CB) samples were included in the study. All patients were diagnosed in Hematology Clinic, Istanbul Medical Faculty, Istanbul University (Table 1). Informed consents were obtained according to the guidelines outlined by the Ethical Review Board of the Istanbul Medical Faculty, Istanbul University with 105906 approval number.

**Table 1:** Clinical characteristics of the Ph- MPN patients enrolled to the study.

Patient No.	First-line treatment	Current				Thrombosis History	Sex	Age	Age of diagnose	Diagnose	*JAK2*V617F allele status
		HGB (g/dL)	HCT (%)	WBC (10^3^ mm^3^)	PLT (10^3^/uL)						
P7	P-H-A	12.70	38	39	162.0	NA	M	64	59	PMF	Monoallele
P8	P-H-A	14	41,6	8.2	83.0	N	F	65	57	PV	Monoallele
P10	P-A	16.5	48.3	9.8	241.0	N	M	32	21	PV	Wild type
P11	P-H-A	12.70	38	39	162.0	NA	M	64	59	PMF	Monoallele
P15	P-H-A	13.4	38	8.8	405.0	N	F	72	63	PV	Monoallele
P23	P-H-V	14.9	48	11.6	229.0	Y	F	63	35	PV	Monoallele
P24	P-A	15.8	47	5.7	277.0	N	M	67	54	PV	Wild type
P26	P-H-T-U-A	14.4	44.6	6.6	646.0	Y	F	75	55	ET	Monoallele
P27	P-H-A-f	12.8	38.4	4.8	506.0	N	F	55	40	ET	Monoallele
P29	P-H-A-U	15.3	48.8	9.1	206.0	N	M	89	67	PV	Monoallele
P33	P-A	16.8	53.6	7.9	685	Y	M	63	63	PV	Wild type
P34	P- H- A	13.2	42.5	9.1	241.0	Y	M	62	56	PV	Wild type
P35	P-H-A-U	14.8	44	6.09	200.0	N	M	75	68	PV	Wild type
P39	P-H-A	14.7	48.8	7.8	453.0	Y	M	46	41	PV	Wild type
P64	P-H-U’-D	15.8	50	20.0	126.0	N	F	56	38	PV, Budd Chiari	Bi-allele
P66	P-H	18.5	53.4	10.6	156.0	N	M	45	37	PV, PMF	Bi-allele
P68	P-H-U	14.9	48	5.3	238.0	N	F	46	36	PV, PMF	Bi-allele
P69	P- H-A	16.3	49	6.2	220.0	N	M	49	32	ET	Bi-allele

Abbreviations: Ph^-^: Philadelphia chromosome negative; HGB: hemoglobin, HCT: hematocrit, WBC: white blood cells; PLT: platelet; P: Phlebotomy, H: Hydroxyurea, T: Thrombo-reductin, U: Uricolysis, U’: Ursodeoxycholic acid, D: Diclofenac sodium A: Acetylsalicyclic acid, V: Varfarin f: Folbiol, NA: not available, N: none; Y: yes; F: female, M: male; PV:Polycythemia vera; PMF: primary myelofibrosis; ET: essential thrombocyhemia:.

### Isolation, labeling, and sungroup sorting of mononuclear cells

Mononuclear cells (MNCs) were isolated by Ficoll gradient centrifugation (Ficoll-Paque premium; GE Healthcare, Upsala, Switzerland) from peripheral blood of patients with MPN after phlebotomy and from CB samples. The collected MNCs were counted and resuspended in a PBS containing 7.5 % BSA + 0.5 mM EDTA (hereafter referred to as "buffer").

The sorting of MNC subgroups were explained elsewhere [36]. Briefly, the CD45 negative (CD45^-^) population were sorted into four different quadrants including HSCs and multipotent progenitors (MPPs) populations that were enriched with CD34^+^CD133^+^ cells, erythro‑myeloid-restricted progenitors (EMPs) that were enriched with CD34^+^CD133^-^ cells, endothelial progenitor cells (EPCs) and HSCs were enriched with CD34^-^CD133^-^cells, and EPCs population for CD34^-^ CD133^+^cells. The CD45^-^ population of MNCs of four MPN patients (P11, P15, P23 and P29) were further sorted according to PAR^±^ and CD34^±^ cell surface expressions using AlexaFluor488-anti-PAR1 (FAB3855 G,R&D Biosystems, MN, USA) and PE-anti-CD34 (Clone 8G12, BD Biosciences, San Jose, CA, USA) with 0.1 % propidium iodide in buffer.

In some analyses, in addition to, cell sorting the MNCs were subjected to CD45^-^ CD34^+/ depleted^ separation process using a magnetic separator columns (LS, Miltenyi Biotec, Bergisch Gladbach, Germany) with magnetic microbeads conjugated to CD45 and CD34 antibodies (130-045-801 and 130-046-702 respectively, Miltenyi Biotec, Bergisch Gladbach, Germany) were applied according to the manufacturer's instructions. Two separate groups, including CD45^-^CD34^+^ and CD45^-^CD34^depleted^ cells were acquired. The purity of the obtained cells was analyzed with flow cytometry (FACSCalibur, BD Bioscience, San Jose, CA, USA) with range of >80-85 %. The intra cytoplasmic detection of phospho-Tyr694 on STAT5 were performed with IntraPrep Permeabilization Reagent (A07803, Beckman Coulter Life Sciences; Indianapolis, Indiana, USA) as suggested by the manufacturer with mAb for APC- anti-p-STAT5 (BioLegend, San Diego, California, USA).

### The use of thrombin and inhibitors in cell culture

The MNCs and/or CD45^-^CD34^+/depleted^ cells of MPN patients or healthy volunteers were incubated with the combination of varying treatments including thrombin (Sigma Aldrich, St. Louis, Missouri, USA), selective PAR1 antagonist, Vorapaxar (SCH530348, MedChemExpress, Monmouth Junction, NJ, USA) and JAK2 inhibitor, Ruxolitinib (sc-364729, Santa Cruz Biotechnology, Dallas, Texas, USA) for the analysis. For all MNC samples, thrombin (1-10 U) [[37], [38], [39], [40]], vorapaxar (80 µM) [41,42], and ruxolitinib (300 nM) were chosen on the basis of the literature and our previous experiences. The cells were cultured with 500,000 cells/well and 12hour (h) of serum-free medium [43]. Treatment with ruxolitinib was performed for 3 h for biallelic *JAK2*V617F and 1.5 h for non-mutant or monoallelic *JAK2*V617F, followed by incubation with vorapaxar for 10 min [44]. After adding thrombin and extending the incubation period to 1 h, the expression of proteins and mRNAs were examined.

### Mutation analysis by allele-specific nested PCR

Genomic DNA was extracted using PureLink Genomic DNA Mini Kit (Invitrogen,) according to the manufacturer's instructions. The *JAK2*V617F mutation was detected by nested allele-specific PCR in all MPN patients and sorted compartments of MPN patients as described previously [45]. Agarose gel electrophoresis revealed a band for a mutant allele of *JAK2*V617F of 279 base pairs (bps) and a wild-type allele of *JAK2* of 229 bps, shown with a 50 bp ladder (Bio-Rad, Hercules, California, USA).

### RNA isolation and real-time quantitative RT-PCR

Total RNA was extracted using a Pico Pure RNA Extraction Kit (Applied Biosystems, USA). First-strand cDNA was synthesized from 60 ng of total RNA using the SCRIPT cDNA Synthesis Kit (Jena Bioscience GmbH, Dortmund, Germany) as per manufacturer's instructions. Gene expression differences were detected using Universal Probe Library (UPL) probes (Roche, Switzerland). For PAR1 expression, primers for the ENST00000319211.4 transcript variant were paired with UPL qPCR Probe #17. Beta-actin (ACTB) served as the reference gene with NM_001101.3 transcript variant and UPL probe #64. Reactions were performed in 20 µl volumes using 1X ORA qPCR Probe Mix (HighQu GmbH, Germany) and analyzed on a LightCycler 480 (Roche Diagnostics, USA).

### Expression analysis of PAR pathway related genes

A gene expression panel of PAR pathway containing a total of 84 genes was performed using the RT^2^ Profiles PCR Array (PAHZ-159; Qiagen GmbH, Düsseldorf, Germany). A startup of RNA (60 ng) using the RNeasy MicroPlus Kit (Qiagen GmbH, Düsseldorf, Germany) was applied for cDNA preparations. The gene expressions were detected with SYBR green technology by a real-time PCR instrument. The expression levels of each gene were calculated using the online service provided by the company.

### Statistical analysis

Gene expression fold changes were determined using the arithmetic mean of C_T_s values, with each experiment performed twice in duplicateRelative *PAR1* expression levels were calculated using the 2^-∆Ct^ formula= 2^- (CTExample – CTReferance)^ with *ACTB* as the reference gene. For drug combination experiments with patients and healthy volunteer controls, the 2^-∆∆Ct^ method= 2^- (∆Ct- (CTSample - CTcontrol))^ was used [46]. Statistical analysis was conducted using GraphPad Prism 8.0 (GraphPad Software, San Diego, California USA, www.graphpad.com). The Mann-Whitney U test compared relative mRNA levels between patient and control samples, while the Friedman and Kruskal-Wallis tests were applied for multidrug experiments and protein expression analyses. Mean values, standard deviations (SD), 95% confidence intervals, percentages, and frequencies were reported, with statistical significance noted in each figure legend.

## Results

*PAR1* activation has been linked to key processes such as inflammation, thrombosis, cell migration, and cancer metastasis. Since MPNs originate HSCs, understanding PAR1 activation in HSCs is crucial for uncovering its role in hematopoiesis and its potential contributions to MPN pathogenesis. Therefore, we aimed to elucidate the activation dynamics of the *PAR1* gene in HPSCs from the peripheral blood of MPN patients and to investigate how its expression is modulated in response to external stimuli, such as thrombin.

### *PAR1* expression in HSPCs subpopulations

**Fig. 1:**
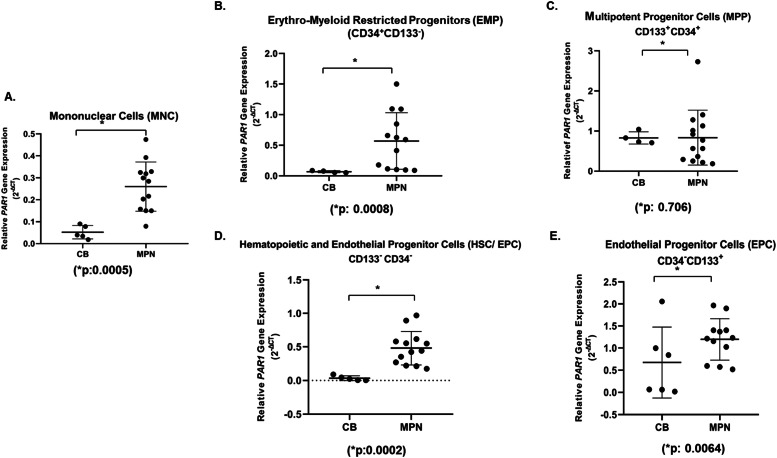
Relative *PAR1* gene expression analysis of (A) mononuclear cells (*p* = 0.0005, Mann-Whitney U), (B) erythro‑myeloid restricted progenitors (EMPs, CD34+CD133-) (*p* = 0.0008, Mann-Whitney U), (C) multipotent progenitor cells (MPPs, CD133+CD34+) (*p* = 0.706, Mann-Whitney U), (D) hematopoietic and endothelial progenitor cells (HSC/ EPC, CD133-CD34-) (*p* = 0.0002, Mann-Whitney U), (E) endothelial progenitor cells (EPCs, CD34-CD133+) (*p* = 0.0064, Mann-Whitney U) in samples from 13 patients with myeloproliferative neoplasms and 5 control samples of cord blood. In each figure, the standard deviation of the mean (SD) is shown with a bar.

*PAR1* activation has been linked to the proliferation of HSPCs. Therefore, an understanding of the level of PAR1 activation in relation to HSPCs subsets in MPNs may be an initial step towards uncovering potential regulatory networks that influence the pro-inflammatory state of MPNs, with implications for both normal hematopoiesis and pathological conditions. *PAR1* gene expression was analyzed using four different HSPC subsets that were identified based on previous studies [36]. HSCs and multipotent progenitors (MPPs) fraction enriched for CD34^+^CD133^+^ cells, erythro‑myeloid-restricted progenitors (EMPs) enriched for CD34^+^CD133^-^ cells, endothelial progenitor cells (EPCs) and HSCs enriched for CD34^-^CD133^-^ cells, and EPCs population enriched for CD34^-^ CD133^+^cells. The initial analysis of *PAR1* expression was carried out on samples of MNCs from 13 patients with MPN and 5 CB. (Fig.1). The relative *PAR1* expression levels in MNCs isolated from MPN patients and CB samples revealed that compared to CB, MNCs from MPN patients were found to have higher relative *PAR1* expression level (*p* = 0.0005) (Fig. 1A). Detailed analysis of *PAR1* in HPSCs subsets of MPN patients showed that the relative *PAR1* expression was increased in EMP (*p* = 0.0008), HSC/EPC (*p* = 0.0002) and EPCs (*p* = 0.0064) of MPN patients compared to CB (Fig.1B, D and E respectively). Surprisingly, the MPPs did not show a significant change in the relative *PAR1* expression compared to the CB (*p* = 0.7) (Fig. 1C).

### *PAR1* expression of MPN samples with *JAK2*V617F allele status

The demonstration of a significant increase in *PAR1* expression in peripheral blood MNCs and HSPCs from MPN patients raises the possibility that *JAK2*V617F plays a role in this activation. Therefore, the comparative analysis of the *JAK2*V617F allele status of HSPCs subsets and sorted populations of PAR1^±^CD34^±^in MPN samples is crucial to gain insight into the hierarchy of PAR1 involvement in *JAK2*V617F activation across the cellular pool and within specific subsets.

**Fig. 2:**
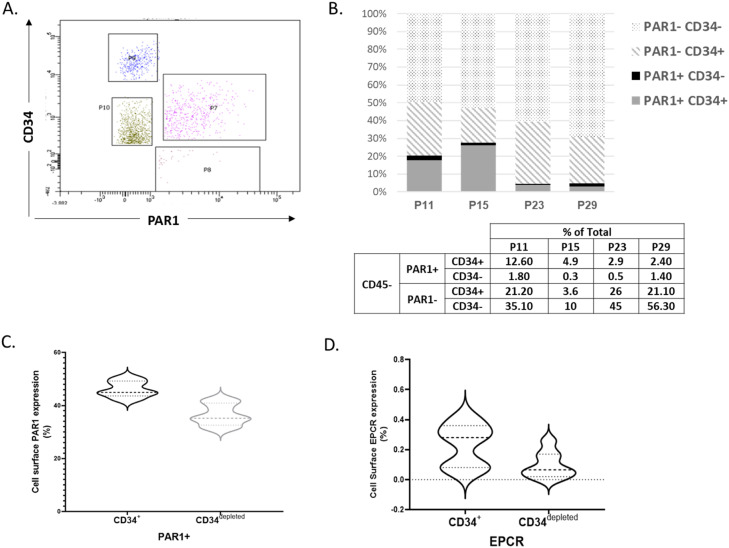
PAR1 cell surface expression analysis of MPN cells (4 MPN patients with monoallelic JAK2V617F) (A) fluorescence-activated cell sorting plot with CD34 and PAR1 expression, (B and C) cell sorting according to CD34 and PAR1 expression that lack of CD45 expression. (D) EPCR cell surface expression analysis of MPN cells with CD34 expression.

The *JAK2*V617F allele status was tested in MNCs and sorted populations of PAR1^±^CD34^±^, EPC, HSC/EPC EMP and MPP from four MPN patients (Table 2). The four selected MPN patients (P11, P15, P23, and P29) who had monoallelic *JAK2*V617F in their MNCs showed that their PAR1^±^CD34^±^ sorted populations also carried monoallelic *JAK2*V617F. Surprisingly, the two MPN patients (P15 and P23) without mutation in their MPPs, showed monoallelic *JAK2*V617F status in their PAR1^±^CD34^±^ populations and in the remaining peripheral blood HSPC subsets including EMPs, HSC/EPCs and EPCs. This finding may indicate that the level of activation of PAR1 is at a more differentiated stage than that of *JAK2*V617F in relation to the appearance of *JAK2*V617F. Subsequently, the PAR1 cell surface expression analysis of MPN cells with CD34 expression (Fig. 2A) revealed that most of the PAR1^+^population also express CD34^+^ (Fig. 2B and C) and still there is a smaller population of PAR1^+^cells which does not express CD34 in a population that lacks CD45 expression (Fig. 2B). On the other hand, the CD34^depleted^ MPN cells revealed ∼40 % PAR1 cell surface expression in CD45^-^ compartment. In addition, EPCR expression were ∼0.4 % in CD34^+^ cells and ∼ 0.2 % in CD34^depleted^ CD45^-^ population (Fig. 2C).

**Table 2:** JAK2V617F allele status of Mononuclear Cells and PAR1 sorted populations of Ph- MPN samples.

Patient No.	MNC	CD133+		CD133-		PAR^+^		PAR^-^	
		CD34+	CD34-	CD34+	CD34-	CD34^+^	CD34^-^	CD34+	**CD34-**
P11	**Monoallele**	Monoallele	Monoallele	Monoallele	Monoallele	**Monoallele**	**Monoallele**	**Monoallele**	**Monoallele**
P15	**Monoallele**	Wild Type	Monoallele	Monoallele	Monoallele	**Monoallele**	**Monoallele**	**Monoallele**	**Monoallele**
P23	**Monoallele**	Wild Type	Monoallele	Monoallele	Monoallele	**Monoallele**	**Monoallele**	**Monoallele**	**Monoallele**
P29	**Monoallele**	Monoallele	Monoallele	Monoallele	Monoallele	**Monoallele**	**Monoallele**	**Monoallele**	**Monoallele**

### Targeting the PAR1 activation: the effect PAR1 antagonists on CD45^-^CD34^+/depleted^ cells

Investigating the correlation between *JAK2*V617F and *PAR1* expression profiles in sorted populations provided some insight into the functional consequences and complex interplay between these factors in the context of MPNs. The significantly active *PAR1* expression in HSPCs subsets might be a therapeutic target which will help in the development of targeted interventions to improve clinical outcomes in MPN cases. We, therefore applied a PAR1 antagonists (vorapaxar) to isolated MPN cells from CD45^-^CD34^+/depleted^ populations and MNCs. The initial cell culture parameters were set to mimic the *in vivo* environment of MPNs, which are known to have elevated levels of thrombin. To reduce baseline signaling activation before treatments, cells were first serum-starved for 12 h. Following starvation, cells were incubated with thrombin for 1 h to activate PAR1 expression. For inhibition studies, cells were treated with the PAR1 antagonist vorapaxar (80 µM) for 10 min before thrombin addition. To assess the combined effect of JAK2 inhibition, ruxolitinib (300 nM) was applied for 3 h in biallelic JAK2V617F cases and 1.5 h in monoallelic JAK2V617F or wild-type JAK2 cells, prior to vorapaxar and thrombin treatments. Following treatments, cells were collected for qRT-PCR and flow cytometry analysis to assess PAR1 expression and related signaling pathways.

**Fig. 3:**
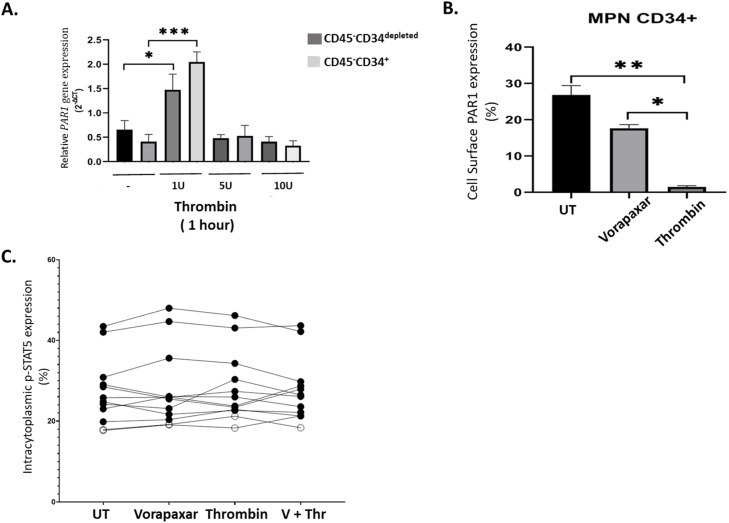
The effect of thrombin on *PAR1* expression was evaluated in (A) CD34^+/depleted^ cells who had lack of CD45 expression (B) the inhibitory effects of vorapaxar were tested in PAR1 cell surface expression (C) and on the phosphorylation of STAT-5B. GraphPad Prism 8.0 software was used for the statistical analysis. (Friedman Test; mean ± SEM, *P < 0.05, **P < 0.01, **P < 0.001).

In order to assess the effect of thrombin on *PAR1* expression, we evaluated the varying thrombin concentrations of 1, 5, and 10 U for 1 h of incubation on CD45^-^CD34^+^and CD45^-^CD34^depleted^ populations. It revealed that thrombin of 1 U had a statistically significant effect on the upregulation of *PAR1* gene expression in both the CD45^-^CD34^+^and CD45^-^CD34^depleted^ populations (*p* = 0.005) and surprisingly, higher concentrations of thrombin tested, 5 U and 10 U, had no effect on *PAR1* expression (Fig. 3A).

Following the activation on *PAR1* expression with thrombin (1U), the inhibitory effects of the PAR1 antagonists, vorapaxar (80 µM) were tested on the cultured MPN CD45^-^CD34^+/depleted^ populations. Since a non-hemostatic, platelet-independent inhibitor, vorapaxar blocks the thrombin binding to the PAR1 receptor, the expression of cell surface PAR1 expression (%) in MPN CD34^+^ cells were determined to be significantly reduced after vorapaxar treatment (Fig. 3B). Thrombin itself cleaves the PAR1 receptor therefore the cell surface expression of PAR1 is diminished after thrombin (1U) treatment (*P < 0.05, **P < 0.01) (Fig. 3B).

The effect of thrombin and vorapaxar application to the intracytoplasmic STAT5B phosphorylation was determined with flow cytometry analysis and revealed that the vorapaxar application did not have any significant effect on the phosphorylation of STAT-5B (Fig. 3C).

### The effect JAK2 inhibitor and Ruxolitinib on PAR1 activation

**Fig. 4:**
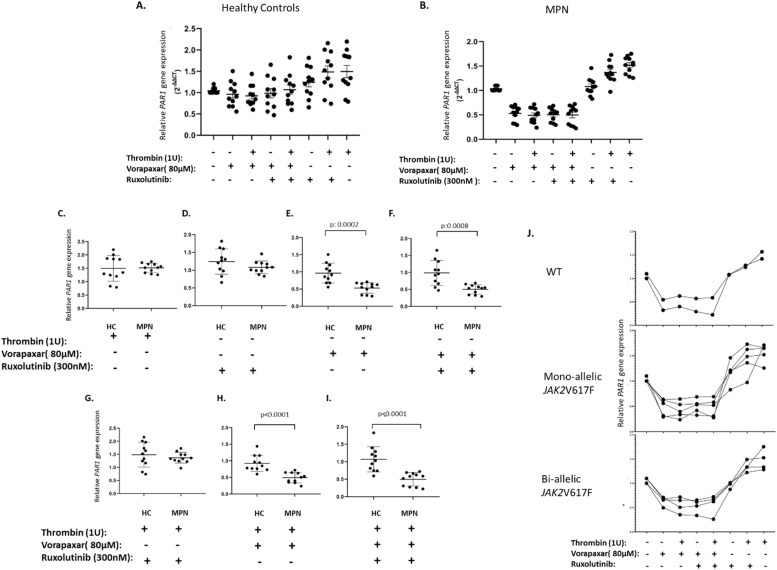
The effect of PAR1 activation with thrombin in JAK2 pathway. Mononuclear cells obtained from MPN patients (n: 11) and healthy controls (n: 11) were cultured overnight in RPMI 1640 serum-free conditions and then treated with Ruxolitinib (300 nm, 3 h (monoallelic JAK2V617F) or 1.5 h (biallelic JAK2V617F and wild type), thrombin (1U, 1 h), vorapaxar (80uM, 1 h) to perform relative PAR1 gene expression analysis (A-I). In patients with monoallelic JAK2V617F (n: 4), biallelic JAK2V617F (n: 5) and wild type (n: 2), the effects of thrombin, vorapaxar and ruxolitinib administration on relative PAR1 gene expression were analyzed (J).

Understanding the molecular mechanisms underlying the activation of the *PAR1* and its interplay with the *JAK2*V617F*,* is critical for the development of targeted therapeutic strategies for MPNs. Therefore, the effect of *PAR1* activation with thrombin in JAK2 pathway is further investigated by PAR1 inhibitor combined with JAK2 tyrosine kinase inhibitor Ruxolitinib. The combined effect of the ruxolitinib and vorapaxar were determined with MNCs of patients with MPN (n:11) and healthy volunteers (n:11). The MNCs were incubated with PAR1 activator thrombin (1U), PAR1 inhibitor vorapaxar (80 µM) and JAK inhibitor Ruxolitinib (300 nM) and their various in order to understand their effects on *PAR1* gene expressions (Fig. 4A and B). *PAR1* expression was activated in the presence of 1 U thrombin in healthy volunteers and MPN patients compared to untreated controls (Fig. 4A, B) and did not revealed any significant effect between healthy volunteers and patients with MPN (Fig. 4C). However, Ruxolitinib, which did not reveal and significant effect on *PAR1* expression between MPN to healthy volunteers (Fig. 4A, B and D) was inhibited *PAR1* expression significantly in the presence of a vorapaxar (p:0.0008) (Fig. 4F). When the combined effects of administrations were compared with untreated MNCs vorapaxar combined applications were significant in MPN cells (Fig. 4A and B). Furthermore, vorapaxar administration was significantly downregulated *PAR1* expressions in MPN patients compared to healthy volunteers (Fig. 4E, F, H and I) (*p* < 0.0001). The detailed analysis of MPN patients regarding to their *JAK2*V617F allele status and the *PAR1* expression were performed including four patients carrying monoallelic *JAK2*V617F, five patients bi-allelic *JAK2*V617F and two wild type JAK2 patients and did not revealed any significant difference in a such comparison (Fig. 4J).

**Fig. 5:**
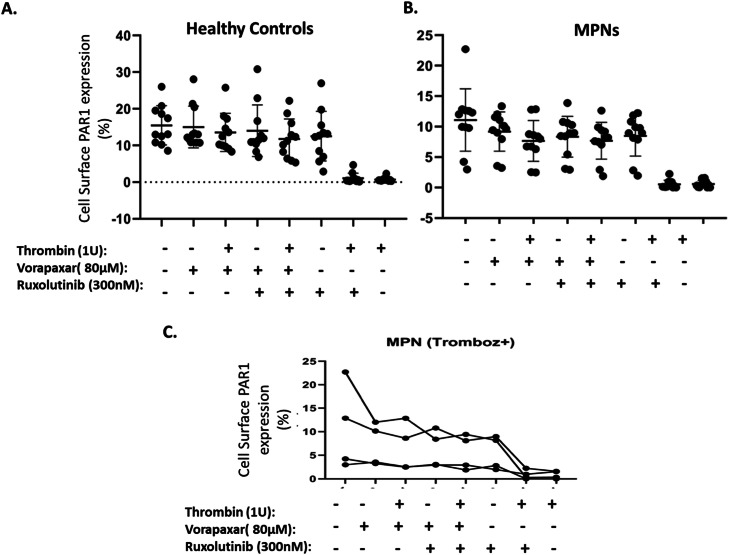
The cell surface PAR1 expression pattern evaluated after treated MNC cells with thrombin (1U), vorapaxar (80uM), ruxolitinib (300 nm) healthy controls (n:11, *p* < 0.0001) (A), MPN patients (n:11, *p* < 0.0001) (B) and MPN patients have history of thrombosis (n:4, p:0.001) (C). Results are performed on two separate occasions. GraphPad Prism 8.0 software was used for the statistical analysis of cell surface protein expression data. (Friedmann Test).

The cell surface PAR1 expressions were analyzed within the same settings of applications to the MNCs of healthy volunteers and MPN patients and did not revealed any significant difference in between (Fig. 5A and B). Since the thrombin cleaves the PAR1 receptor, cell surface PAR1 expressions were diminished with combinations of thrombin applications (Fig. 5A and B). Furthermore, four MPN patients that have history of thrombosis revealed similar *PAR1* expression pattern in applications (Fig. 5C).

### Expression changes of PAR pathway related genes

**Fig. 6:**
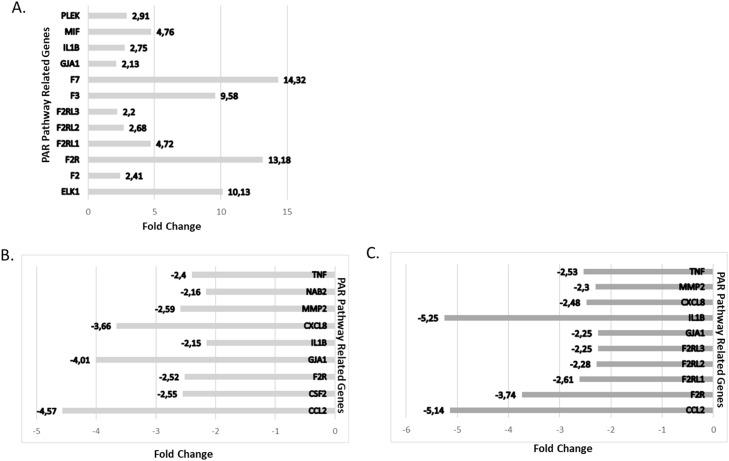
The responses of MPN cells to PAR1 inhibition by detection of changes in PAR pathway. (A) Jak2V617F-induced PAR regulated genes and (C) trombin-PAR-coagulation suppressed genes affected >2-fold in the PAR pathway in mononuclear cells of MPN patients (2 patients with monoallelic JAK2V617F (p26 and p:29), 2 patients with biallelic JAK2V617F (p:64 and p:68) and no mutation carrying one patient (p: 35)) by PAR1 inhibition. (B) PAR pathway-related genes downregulated >2-fold in one MPN patient with history of thrombosis (p:26).

By deciphering the molecular events associated with PAR1 activation, we pursue to uncover potential regulatory networks that influence the responses of MPN cells to *PAR1* inhibition by detection of changes in PAR pathway related genes. In this way, the thrombin activated PAR pathway was analyzed within 84 different genes and gene expression changes were determined in MNCs of MPN patients. In the study, gene expression changes of two patients with monoallelic *JAK2*V617F (p26 and p29), one of which is known to have a history of thrombosis, two patients with biallelic *JAK2*V617F (p64 and p68), and one patient with no *JAK2*V617F mutation (p35) were analyzed in the panel (Fig. 6). The comparison of gene expressional fold changes of MNCs revealed that the *JAK2*V617F induce PAR-regulated genes including *PLEK, MIF, IL-1B, GJA1, F7, F3 (*Tissue Factor*), F2RL3 (PAR4), F2RL2 (PAR3), F2RL1 (PAR2), F2R (PAR1), F2 (*Thrombin*)* and *ELK1* >2 fold (Fig. 6A). The effect of vorapaxar administration to the PAR-regulated genes were analyzed in MNCs of MPN patient (P26) with a history of thrombosis and compared to the untreated samples and normalized to controls in the panel (Fig. 6B). The expression of genes involved in thrombin/PAR signaling pathway was suppressed and genes including *CCL2, CSF2, F2R* (PAR1), *GJA1, IL1B, CXCL8, NAB2, TNF*, and *MMP2*, were decreased >2-fold. This might reveal that PAR1 selective inhibition of vorapaxar and other inhibited genes in this pathway are also involved in thrombin and coagulation signaling in relation to PAR1. Vorapaxar showed a similar mechanism of action in all other three patients and was effective on downregulation of gene expressions in PAR/ thrombin/ coagulation pathway (Fig. 6C).

## Discussion

In this study, we analyzed the expression and activation of PAR1 in HPSCs from patients with Ph^-^ MPNs, investigating the interaction between thrombin-induced PAR1 activation and the *JAK2*V617F mutation. Our results demonstrate a pronounced upregulation of *PAR1* expression, especially in cells bearing the *JAK2*V617F mutation, which is known to activate various signaling pathways contributing to the pathological characteristics of MPNs, such as thrombosis and inflammation [47,48].

We highlighted the potential interactions between PAR1 and JAK2, suggesting that these interactions may contribute to the hypercoagulable state observed in MPNs. Notably, our findings indicate that *PAR1* gene expression is elevated in specific subsets of peripheral blood HPSCs, including endothelial EPCs, HSC/EPCs, and EMPs, but not in MPPs. This suggests a stage-specific regulation of PAR1 activation, which becomes more pronounced as cells continue to differentiate. Furthermore, the presence of monoallelic *JAK2*V617F mutations in PAR1^±^CD34^±^populations and other peripheral blood HSPC subsets, excluding MPPs, suggests that *PAR1* activation could occur at a more advanced stage of differentiation compared to the onset of the *JAK2*V617F mutation. This finding highlights a complex regulatory mechanism that influences these markers at different stages of cellular development, thereby impacting the thrombo-inflammatory responses that are central to the pathogenesis of MPNs. Additionally, increased HSPCs mobilization in MPNs, contrasted with normal conditions, remains poorly understood. Studies have shown that mice lacking PAR1 exhibit improved HSC retention in the bone marrow, whereas thrombin-PAR1 signaling enhances nitric oxide (NO) production, leading to HSC regress from the bone marrow. Our previous research indicated a significant increase in endothelial nitric oxide synthase expression in *JAK2*V617F^+^CD34^+^ cells under both normoxic and hypoxic conditions [49], which may have related to the elevated *PAR1* expression in these cells. The importance of chronic inflammation in thrombosis has been well documented [50]. It is generally thought that thrombin activation of PAR1 initiates proinflammatory signaling on vascular ECs [[51], [52], [53]]. However, in malignancies, as we have shown within this study, such activation likely originates from more primitive cells such as EPCs rather than fully differentiated ECs. Also, the EPCR was not detectable at the protein level in CD34^±^ cells, indicating lack of aPC/EPCR-mediated PAR activation. Therefore, the increased expression of *PAR1* suggesting procoagulative and proinflammatory induction remains active.

The role of PAR1 across different cancers, where it acts variably as an oncogene in breast and prostate cancers and is involved in malignancy processes like angiogenesis, tumor invasiveness, and metastasis, is well documented [[54], [55], [56]]. In contrast, PAR1 expression has been shown to be lower in AML CD34^+^blast cells [57] highlighting potential differences in its regulatory mechanisms between AML and MPNs. Given the distinct disease courses and hematological profiles of these disorders, factors such as platelet activity, inflammatory signaling, and disease chronicity may differentially influence PAR1-related pathways. Further research is warranted to explore the implications of these differences in the broader context of hematologic malignancies. In addition, inhibition of PAR1 has impeded the progression of several tumor types, including melanoma, colon cancer, and pancreatic cancer [58].

We have investigated the effects of thrombin-induced PAR1 activation within the JAK2 signaling pathway, utilizing a combination of the PAR1 inhibitor-vorapaxar and the JAK2 tyrosine kinase inhibitor- ruxolitinib. We have employed PAR1 inhibition as a new therapeutic option in MPNs, underscoring the importance of targeting uncommitted hematopoietic progenitors responsible for the initiation of these diseases, which may involve PAR signaling pathways. Experimental results both MPNs and healthy volunteers reveal that while thrombin alone did not significantly alter *PAR1* expression, the use of ruxolitinib in conjunction with vorapaxar significantly inhibited *PAR1* expression. Notably, vorapaxar substantially reduced *PAR1* levels in MPN patients compared to controls, highlighting its potential as a therapeutic agent. This combination therapy appears particularly effective in MPN cells, irrespective of *JAK2*V617F allele status, suggesting a promising strategy for mitigating disease progression through targeted molecular inhibition.

PAR1 activation stimulates the release of cytokines and growth factors, pivotal in sustaining and promoting the proliferation of HSPCs. Activated PAR1 also modulates the expression of key transcription factors that regulate hematopoiesis, influencing the differentiation and fate of HSCs and progenitor cells. Surprisingly, several PAR-pathway-related genes, including *CCL2, CSF2, GJA1, IL1B, CXCL8, NAB2, TNF,* and *MMP2*, which play roles in thrombo-inflammation, were significantly downregulated (>2-fold) after treatment. Suggesting that PAR1 activation might be a critical regulator in thrombo-inflammatory and fibrotic complications detected in MPNs. The constitutively active JAK/STAT pathway in MPNs may lower the threshold for thrombin-induced PAR1 activation, leading to an exaggerated inflammatory response and a procoagulant state.

Prothrombotic events in MPNs are influenced by factors such as erythrocytosis, leukocytosis, and platelet activation. Given the central role of platelets in thrombin-induced aggregation and thrombosis, which is mediated through PAR1, this identifies a crucial gap in our current study. Future research will aim to extend our understanding of thrombotic mechanisms in MPNs and explore how they are influenced by PAR1 inhibition. This additional study is essential for developing more effective therapeutic strategies targeting the intricate balance of clotting and bleeding risks in MPN patients. Previous studies have shown that vorapaxar does not significantly affect clot viscoelasticity, EC function, or fibrin formation via ADP-induced pathways. It also had no impact on coagulation markers like d-dimer, TT, PT, PTT, fibrinogen, or vWF [59]. PAR-1 inhibition, has also proven effective against atherosclerosis and vascular inflammation [60]. Targeting abnormal PAR1 activation with vorapaxar, the only FDA-approved drug in this category, presents a potential strategy to mitigate MPN progression. However, its use is tempered by safety concerns [61], notably an increased risk of bleeding as demonstrated in trials like TRA2P-TIMI 50 [62] —a critical issue for MPN patients who are predisposed to both thrombotic and bleeding complications. This underscores the need for a careful therapeutic approach that considers the delicate balance required to manage these opposing risks effectively. Our findings revealed increased PAR1 expression in HPSCs subsets and MNCs, which is unique compared to general clinical scenarios involving PAR1 inhibitors. This distinctive overexpression could uniquely influence the pathophysiology of MPNs, suggesting a need to reevaluate the risk-benefit profile of PAR1 inhibition in these patients.

The thrombin activated PAR pathway was analyzed and for the first time in the literature, the effect of PAR1 was functionally demonstrated in the MPNs. Understanding thrombin-induced PAR1 activation and exploring the potential role of JAK2 in such activation, especially in MPNs, could significantly advance our knowledge of the pathophysiology of these disorders and open new avenues for treatment. Still, this area requires further research to establish definitive links and mechanisms.

## Ethics approval and consent to participate

Ethics approval is required for this study and approved by Ethical Review Board of the Istanbul Medical Faculty, Istanbul University.

## Consent for publication

Yes

## Availability of data and material

Not needed

## Funding

This work was supported by the Research Fund of TUBITAK (Project No. 112S483) and The Research Fund of Istanbul University (Project No. TSA-2017-25370 and TDK-2021-38358)

## CRediT authorship contribution statement

**İldeniz USLU-BIÇAK:** Writing – original draft, Validation, Methodology, Investigation, Formal analysis, Data curation. **Meliha NALÇACI:** Supervision, Resources. **Selçuk SÖZER:** Writing – review & editing, Supervision, Resources, Methodology, Conceptualization.

## Declaration of competing interest

The authors declare that they have no known competing financial interests or personal relationships that could have appeared to influence the work reported in this paper. The author is an Editorial Board Member/Editor-in-Chief/Associate Editor/Guest Editor for *[Journal name]* and was not involved in the editorial review or the decision to publish this article.

## References

[1] Mead A.J., Mullally A. (2017). Myeloproliferative neoplasm stem cells. Blood.

[2] Tefferi A., Vainchenker W. (2011). Myeloproliferative neoplasms: molecular pathophysiology, essential clinical understanding, and treatment strategies. J. Clinic. Oncol..

[3] Hasselbalch H.C. (2013). Chronic inflammation as a promotor of mutagenesis in essential thrombocythemia, polycythemia vera and myelofibrosis. A human inflammation model for cancer development?. Leuk. Res..

[4] Marchioli R., Finazzi G., Specchia G., Cacciola R., Cavazzina R., Cilloni D. (2013). Cardiovascular events and intensity of treatment in polycythemia vera. New England J. Med..

[5] Barbui T., Finazzi G., Falanga A. (2013). Myeloproliferative neoplasms and thrombosis. Blood, J. Am. Soc. Hematol...

[6] Barosi G. (2014). An immune dysregulation in MPN. Curr. Hematol. Malig. Rep..

[7] Barbui T., Carobbio A., Finazzi G., Vannucchi A.M., Barosi G., Antonioli E. (2011). Inflammation and thrombosis in essential thrombocythemia and polycythemia vera: different role of C-reactive protein and pentraxin 3. Haematologica.

[8] Finazzi G., De Stefano V., Barbui T. (2013). Are MPNs vascular diseases?. Curr. Hematol. Malig. Rep..

[9] Schafer A.I. (2021). Thrombotic, vascular, and bleeding complications of the myeloproliferative neoplasms. Hematol. Oncol. Clin. North Am..

[10] Trinchieri G. (2012). Cancer and inflammation: an old intuition with rapidly evolving new concepts. Annu. Rev. Immunol..

[11] Frederiksen H., Farkas D.K., Christiansen C.F., Hasselbalch H.C., Sørensen H.T. (2011). Chronic myeloproliferative neoplasms and subsequent cancer risk: a Danish population-based cohort study. Blood J. Am. Soc. Hematol..

[12] Hasselbalch H.C. (2014). Perspectives on the impact of JAK-inhibitor therapy upon inflammation-mediated comorbidities in myelofibrosis and related neoplasms. Expert. Rev. Hematol..

[13] Kristinsson S.Y., Landgren O., Samuelsson J., Björkholm M., Goldin L.R. (2010). Autoimmunity and the risk of myeloproliferative neoplasms. Haematologica.

[14] Wolach O., Shacham Abulafia A. (2021). Can novel insights into the pathogenesis of myeloproliferative neoplasm-related thrombosis inform novel treatment approaches?. Haematologica.

[15] Bahou W.F., Nierman W.C., Durkin A.S., Potter C.L., Demetrick D.J. (1993). Chromosomal assignment of the Human thrombin receptor gene: localization to region ql3 of chromosome 5. Blood.

[16] Lin H., Liu A.P., Smith T.H., Trejo J. (2013). Cofactoring and dimerization of proteinase-activated receptors. Pharmacol. Rev..

[17] Wojtukiewicz M.Z., Hempel D., Sierko E., Tucker S.C., Honn K.V. (2015). Protease-activated receptors (PARs)—Biology and role in cancer invasion and metastasis. Cancer Metastasis Rev..

[18] Pompili E., Fabrizi C., Fornai F., Fumagalli L. (2019). Role of the protease-activated receptor 1 in regulating the function of glial cells within central and peripheral nervous system. J. Neural Transm..

[19] McEachron T.A., Pawlinski R., Richards K.L., Church F.C., Mackman N. (2010). Protease-activated receptors mediate crosstalk between coagulation and fibrinolysis. Blood, J. Am. Soc. Hematol..

[20] Vu T.-K.H., DT Hung, Wheaton V.I., Coughlin S.R. (1991). Molecular cloning of a functional thrombin receptor reveals a novel proteolytic mechanism of receptor activation. Cell.

[21] Scarborough R., Naughton M., Teng W., Hung D., Rose J., Vu T. (1992). Tethered ligand agonist peptides. Structural requirements for thrombin receptor activation reveal mechanism of proteolytic unmasking of agonist function. J. Biol. Chem..

[22] Andersen H., Greenberg D.L., Fujikawa K., Xu W., Chung D.W., Davie E.W (1999). Protease-activated receptor 1 is the primary mediator of thrombin-stimulated platelet procoagulant activity. Proc. Natl. Acad. Sci..

[23] Fujimoto D., Hirono Y., Goi T., Katayama K., Matsukawa S., Yamaguchi A. (2010). The activation of Proteinase-activated Receptor-1 (PAR1) mediates gastric cancer cell proliferation and invasion. BMC Cancer.

[24] Tsopanoglou N.E., Maragoudakis M.E. (2004). Role of thrombin in angiogenesis and tumor progression. Semin. Thrombosis Hemostasis;.

[25] Gur-Cohen S., Itkin T., Chakrabarty S., Graf C., Kollet O., Ludin A. (2015). PAR1 signaling regulates the retention and recruitment of EPCR-expressing bone marrow hematopoietic stem cells. Nat. Med..

[26] Gur-Cohen S., Kollet O., Graf C., Esmon C.T., Ruf W., Lapidot T. (2016). Regulation of long-term repopulating hematopoietic stem cells by EPCR/PAR1 signaling. Ann. NY Acad. Sci..

[27] Basu S., Liang H.P.H., Hernandez I., Zogg M., Fields B., May J. (2020). Role of thrombomodulin expression on hematopoietic stem cells. J. Thromb. Haemost..

[28] Zini R., Guglielmelli P., Pietra D., Rumi E., Rossi C., Rontauroli S. (2017). CALR mutational status identifies different disease subtypes of essential thrombocythemia showing distinct expression profiles. Blood Cancer J..

[29] Panova-Noeva M., Marchetti M., Spronk H.M., Russo L., Diani E., Finazzi G. (2011). Platelet-induced thrombin generation by the calibrated automated thrombogram assay is increased in patients with essential thrombocythemia and polycythemia vera (vol 86, pg 337, 2011). Am. J. Hematol..

[30] Falanga A., Marchetti M., Vignoli A., Balducci D., Russo L., Guerini V. (2007). V617F JAK-2 mutation in patients with essential thrombocythemia: relation to platelet, granulocyte, and plasma hemostatic and inflammatory molecules. Exp. Hematol..

[31] Panova-Noeva M., Marchetti M., Russo L., Tartari C.J., Leuzzi A., Finazzi G. (2013). ADP-induced platelet aggregation and thrombin generation are increased in Essential thrombocythemia and polycythemia Vera. Thromb. Res..

[32] Mihaila R.-G. (2017). Thrombin generation-a potentially useful biomarker of thrombotic risk in Philadelphia-negative myeloproliferative neoplasms. Biomed. Papers.

[33] Doller D., Chackalamannil S., Czarniecki M., McQuade R., Ruperto V. (1999). Design, synthesis, and structure-activity relationship studies of himbacine derived muscarinic receptor antagonists. Bioorg. Med. Chem. Lett..

[34] Kalantzi K.I., Tsoumani M.E., Goudevenos I.A., Tselepis A.D. (2012). Pharmacodynamic properties of antiplatelet agents: current knowledge and future perspectives. Expert. Rev. Clin. Pharmacol..

[35] Vardiman J.W., Thiele J., Arber D.A., Brunning R.D., Borowitz M.J., Porwit A. (2009). The 2008 revision of the World Health Organization (WHO) classification of myeloid neoplasms and acute leukemia: rationale and important changes. Blood.

[36] İ Uslu Bıçak, B Tokcan, Yavuz A.S., Tokdemir S.S (2023). Circulating CD133+/–CD34– Have increased c-MYC expression in myeloproliferative neoplasms. Turk. J. Haematol..

[37] Schuepbach R.A., Feistritzer C., Brass L.F., Riewald M. (2008). Activated protein C–cleaved protease activated receptor-1 is retained on the endothelial cell surface even in the presence of thrombin. Blood. J. Am. Soc. Hematol..

[38] Ohshiro K., Bui-Nguyen T.M., Natha R.S.D., Schwartz A.M., Levine P., Kumar R. (2012). Thrombin stimulation of inflammatory breast cancer cells leads to aggressiveness via the EGFR-PAR1-Pak1 pathway. Int. J. Biol. Markers.

[39] Orfeo T., Butenas S., Brummel-Ziedins K.E., Mann K.G. (2005). The tissue factor requirement in blood coagulation. J. Biol. Chem..

[40] Di Serio C., Pellerito S., Duarte M., Massi D., Naldini A., Cirino G. (2007). Protease-activated receptor 1-selective antagonist SCH79797 inhibits cell proliferation and induces apoptosis by a protease-activated receptor 1-independent mechanism. Basic Clin. Pharmacol. Toxicol..

[41] Tang W., Huang B., Wang J., An L., Zhong H., Yang H. (2017). A label-free screening approach targeted protease-activated receptor 1 based on dynamic mass redistribution in living cells. RSC Adv..

[42] Pang J., Hu P., Wang J., Jiang J., Lai J. (2019). Vorapaxar stabilizes permeability of the endothelial barrier under cholesterol stimulation via the AKT/JNK and NF‑κb signaling pathways. Mol. Med. Rep..

[43] Ellinghaus P., Perzborn E., Hauenschild P., Gerdes C., Heitmeier S., Visser M. (2016). Expression of pro-inflammatory genes in human endothelial cells: comparison of rivaroxaban and dabigatran. Thromb. Res..

[44] van den Eshof B.L., Hoogendijk A.J., Simpson P.J., van Alphen F.P., Zanivan S., Mertens K. (2017). Paradigm of biased PAR1 (Protease-Activated Receptor-1) activation and inhibition in endothelial cells dissected by phosphoproteomics. Arterioscler. Thromb. Vasc. Biol..

[45] Sozer S., Ishii T., Fiel M.I., Wang J., Wang X., Zhang W. (2009). Human CD34+ cells are capable of generating normal and JAK2V617F positive endothelial like cells in vivo. Blood Cells Mol. Dis..

[46] Livak K.J., Schmittgen T.D. (2001). Analysis of relative gene expression data using real-time quantitative PCR and the 2(-Delta Delta C(T)) method. Methods.

[47] Masselli E., Pozzi G., Gobbi G., Merighi S., Gessi S., Vitale M. (2020). Cytokine profiling in myeloproliferative neoplasms: overview on phenotype correlation, outcome prediction, and role of genetic variants. Cells.

[48] Wong W.J., Baltay M., Getz A., Fuhrman K., Aster J.C., Hasserjian R.P. (2019). Gene expression profiling distinguishes prefibrotic from overtly fibrotic myeloproliferative neoplasms and identifies disease subsets with distinct inflammatory signatures. PLoS. One.

[49] Soroglu C.V., Uslu-Bicak I., Toprak S.F., Yavuz A.S., Sozer S. (2023). Effect of hypoxia on HIF-1alpha and NOS3 expressions in CD34(+) cells of JAK2V617F-positive myeloproliferative neoplasms. Adv. Med. Sci..

[50] Falanga A., Marchetti M., editors. Thrombosis in myeloproliferative neoplasms. Seminars in thrombosis and hemostasis; 2014: Thieme Medical Publishers. 10.1055/s-0034-1370794 24610470

[51] OSSOVSKAYA V.S., BUNNETT N.W (2004). Protease-activated receptors: contribution to physiology and disease. Physiol. Rev..

[52] Coughlin S.R. (2005). Protease-activated receptors in hemostasis, thrombosis and vascular biology. J. Thrombosis Haemostasis.

[53] Subramaniam S., Ogoti Y., Hernandez I., Zogg M., Botros F., Burns R. (2021). A thrombin-PAR1/2 feedback loop amplifies thromboinflammatory endothelial responses to the viral RNA analogue poly(I:C). Blood Adv..

[54] Simon C., Chagraoui J., Krosl J., Gendron P., Wilhelm B., Lemieux S. (2012). A key role for EZH2 and associated genes in mouse and human adult T-cell acute leukemia. Genes Dev..

[55] Simon J.A., Lange C.A. (2008). Roles of the EZH2 histone methyltransferase in cancer epigenetics. Mutation Res..

[56] Varambally S., Dhanasekaran S.M., Zhou M., Barrette T.R., Kumar-Sinha C., Sanda M.G. (2002). The polycomb group protein EZH2 is involved in progression of prostate cancer. Nature.

[57] Bäumer N., Krause A., Köhler G., Lettermann S., Evers G., Hascher A. (2014). Proteinase-activated receptor 1 (PAR1) regulates leukemic stem cell functions. PLoS. One.

[58] Cisowski J., O'Callaghan K., Kuliopulos A., Yang J., Nguyen N., Deng Q. (2011). Targeting protease-activated receptor-1 with cell-penetrating pepducins in lung cancer. Am. J. Pathol..

[59] Bliden K., Chaudhary R., Kuliopulos A., Tran H., Taheri H., Tehrani B. (2020). Effects of vorapaxar on clot characteristics, coagulation, inflammation, and platelet and endothelial function in patients treated with mono- and dual-antiplatelet therapy. J. Thromb. Haemost..

[60] Friebel J., Moritz E., Witkowski M., Jakobs K., Strässler E., Dörner A. (2021). Pleiotropic effects of the protease-activated receptor 1 (PAR1) inhibitor, Vorapaxar, on atherosclerosis and vascular inflammation. Cells.

[61] Tricoci P., Huang Z., Held C., Moliterno D.J., Armstrong P.W., Van de Werf F. (2012).

[62] Scirica B.M., Bonaca M.P., Braunwald E., De Ferrari G.M., Isaza D., Lewis B.S. (2012). Vorapaxar for secondary prevention of thrombotic events for patients with previous myocardial infarction: a prespecified subgroup analysis of the TRA 2°P-TIMI 50 trial. Lancet.

